# Isolation and Characterization of Lactic Acid Bacteria From “*Trites*” Having the Ability to Produce α-Glucosidase Inhibitors

**DOI:** 10.1155/ijm/8864668

**Published:** 2025-01-07

**Authors:** Edy Fachrial, Ismawati, Afif Pranaya Jati, Titania Tjandrawati Nugroho, Saryono

**Affiliations:** ^1^Doctoral Program of Chemistry, Department of Chemistry, Faculty of Mathematics and Natural Science, Universitas Riau, Pekanbaru, Riau 28293, Indonesia; ^2^Department of Biomedical Sciences, Faculty of Medicine, Dentistry, and Health Sciences, Universitas Prima Indonesia, Medan, Indonesia; ^3^Department of Biochemistry, Faculty of Medicine, Universitas Riau, Pekanbaru, Riau 28293, Indonesia; ^4^Indonesian Society of Bioinformatics and Biodiversity, Malang, Indonesia; ^5^Synthetic Biology Division, Bioinformatics Research Center, Indonesian Institute of Bioinformatics, Malang, Indonesia; ^6^Department of Chemistry, Faculty of Mathematics and Natural Science, Universitas Riau, Pekanbaru, Riau 28293, Indonesia

**Keywords:** *α*-glucosidase inhibitor, lactic acid bacteria, *Pediococcus acidilactici* strain LBSU8, probiotics, whole genomic sequencing

## Abstract

Alpha-glucosidase inhibitors are one of the therapies used for treating type 2 diabetes by inhibiting the absorption of carbohydrates in the gastrointestinal tract. In addition to antimicrobial activity, some probiotic species show *α*-glucosidase inhibitor activity, making them potential alternative therapies for type 2 diabetes. This study aimed to characterize probiotics from “*trites*,” a traditional food from North Sumatra, Indonesia, that exhibit *α*-glucosidase inhibition, potentially useful for type 2 diabetes treatment. The probiotic potential of the isolates was evaluated through antagonistic activity, acid tolerance, bile tolerance, and susceptibility to antimicrobial agents. *α*-Glucosidase inhibition was tested with acarbose as a control. The best-performing isolate, LBSU8, was identified as *Pediococcus acidilactici* through 16S rRNA gene sequencing. Gene analysis using genome sequencing for LBSU8 revealed antimicrobial secondary metabolites, including RiPPs, polyketide, and NRP, while capsular polysaccharide might contribute to its antidiabetic activity. Though no specific *α*-glucosidase inhibitory secondary metabolites were identified, enzymes like dTDP-glucose 4,6-dehydratase, transketolase, and glucose-1-phosphate thymidylyltransferase may contribute to this activity. *P. acidilactici* LBSU8 shows potential as an alternative diabetes therapy in the food and drug industries. Further studies are needed to elucidate the exact mechanism behind its *α*-glucosidase inhibitory activity and to explore its efficacy in clinical settings.

## 1. Introduction

Diabetes is a prominent cause of death and disability around the world, affecting people of all ages and genders. Diabetes is estimated to affect about 1.31 billion people by 2050, with prevalence rates exceeding 10% in two super-regions: North Africa and the Middle East (16.8%) and Latin America and the Caribbean (11.3%) [1]. Indonesia is among the top 10 countries with the highest prevalence of type 2 diabetes mellitus (T2DM) at 10.8% [2]. High direct medical expenses were incurred in Indonesia for treating individuals with T2DM. In 2016, Indonesia spent $576 million on treating T2DM and its complications, with 74% of the expense allocated to managing diabetes-related problems. Individuals who experienced complications spent twice as much (US $930/person/year ± US $1480/person/year) as those who did not (US $421/person/year ± US $745/person/year) [3].

Alpha-glucosidase inhibitors are a distinct category of medication used to treat diabetes. These oral medications are enzyme inhibitors that do not primarily affect the pancreas. They regulate postprandial hyperglycemia by inhibiting the absorption of carbohydrates in the gastrointestinal system [4]. The most often reported adverse effects of *α*-glucosidase inhibitors are gastrointestinal problems. These occur due to the breakdown of undigested carbohydrates by bacteria in the colon, producing an excessive amount of gas. The most often reported adverse effect is flatulence, which occurs in approximately 78% of cases. The occurrence of diarrhea and stomach pain is also possible [5].

Research on using probiotics as an alternative therapy in lowering blood glucose levels is still in its early stages, but several studies have shown potential, especially for *α*-glucosidase inhibition. In the previous research, the *Pediococcus acidilactici* strain DNH16 (PADNH16), which was obtained from the traditional food source “Dali ni Horbo” in North Sumatra, exhibited an in vitro *α*-glucosidase inhibitory activity of 27% [6]. The hypoglycemic activity of PADNH16 was demonstrated by in vivo tests conducted on rats using a T2DM model. Dosing PADNH16 had no effect on serum glutamic oxaloacetic transaminase (SGOT), serum glutamic pyruvic transaminase (SGPT), urea, or creatinine levels, indicating it was safe for liver and kidney function. The lipid profile examination indicated equivalent HDL, LDL, and triglyceride levels to the control group. Pancreatic histology showed no changes to pancreatic β cells after PADNH16 injection [7].

In vitro testing methods commonly used in research must be improved to ensure safety. Previous studies have reported that improperly labeled probiotic products contain genes for producing toxic metabolites or virulence genes, which may pose potential health risks [8]. Several studies report that there is a possibility that probiotics have virulence factors. Imre et al. found that the probiotic yeast *Saccharomyces* can be an opportunistic pathogen [9]. Wassenaar et al. found that probiotic product, Symbioflor2 (DSM 17252), contained *Escherichia coli* with virulence-related genes, but these genes did not result in a pathogenic phenotype [10]. It was noted that the whole genomic sequencing method could identify transferable antibiotic resistance genes and other genes of concern in candidate probiotic strains [11]. Wang et al. found that whole genome analysis is a valuable method for evaluating the quality and safety of probiotic products, as it can identify the presence of genes associated with antimicrobial resistance (AMR), virulence factors, and toxic metabolites, which pose potential health risks [8].

Indonesia is rich in culture, traditions, and diverse culinary delights. Each region has its specialty food. One example is a dish called *trites* from the Karo tribe in Medan, North Sumatra. Some people consider this food quite extreme. *Trites* is a traditional Karo dish made from the partially digested grass found within the stomach of oxen, buffaloes, or cows. In the cow's stomach, grass is digested by microbes under anaerobic conditions. This process can take several hours to more than a day. The rumen is then removed through the slaughter process. After the slaughtering process, this rumen content is carefully collected and separated into a dedicated container for further preparation. Until now, no research has been conducted on the probiotic characteristics of *trites*. The present study aimed to isolate and characterize probiotics isolated from *trites* based on their ability as *α*-glucosidase inhibitors.

## 2. Method

### 2.1. Isolation of Lactic Acid Bacteria (LAB)

One gram of *trites* sample was cultured into 9 mL of sterile De Man, Rogosa, and Sharpe broth and incubated for 18–24 h. Because the sample was derived from the buffalo rumen, an anaerobic environment was selected for incubation to mimic the natural anaerobic conditions in the rumen where native microbiota thrive. To achieve anaerobic incubation, we utilized an anaerobic jar system. After the incubation process, serial dilution was carried out up to a dilution of 10^−7^. Serial dilutions were prepared from the initial sample up to the seventh dilution. Subsequently, 0.1 mL of each dilution was inoculated onto the surface of MRS agar (Himedia) supplemented with 1% CaCO_3_ (Merck). The presence of acid-producing bacteria was indicated by a clear zone surrounding the colonies. Bacterial isolates were then randomly selected and continued with purification by re-streaking into fresh MRS agar media. The LAB isolates were stored in 40% glycerol stock media at −20°C [6].

### 2.2. Biochemical Characterization

The purified colonies were characterized by Gram staining, the TSIA (Triple Sugar Iron Agar) test, and the hemolysis and catalase test. Gram staining is done based on the procedure by Claus [12]. The TSIA test is performed by inoculating the TSIA media (Merck) by piercing through the center of the medium to the bottom of the tube and then scraping the agar surface obliquely and incubating the tube at 35°C–37°C for 18–24 h. A distinct color change was observed in the slant and bottom portions of the media, along with cracks, suggesting gas production and hydrogen sulfide formation [13]. The catalase test was performed based on the procedure used by Goyal et al. [14]. The hemolytic assay was determined using Columbia agar (Himedia) containing 5% (w/v) sheep blood, and Petri dishes were incubated at 37°C for 48 h. After incubation, the isolate's hemolytic activity was evaluated and classified based on the lysis of red blood cells around the colony. A green zone around the colony was considered indicative of *α*-hemolysis, a clear zone around the colony was considered as *β*-hemolysis, and the absence of a zone around the colony was considered as *γ*-hemolysis. Only isolates with *γ*-hemolysis were used in the subsequent analysis [15].

### 2.3. Probiotic Characterization

The probiotic isolate characteristic tests were conducted based on antibacterial activity and tolerance to simulated gastric juices and bile salt. Antibacterial activity was determined using the agar disc diffusion method. From each LAB isolate and indicator bacteria, 4-5 colonies were dissolved into 5 mL of sterile nutrient broth (Merck) and compared with a 0.5 McFarland solution, equivalent to 1.5 × 10^8^ CFU/mL. The indicator bacteria used were *Escherichia coli* ATCC 25922 and *Staphylococcus aureus* ATCC 25923. The indicator bacteria were swabbed onto the nutrient agar surface using a sterile cotton swab. Sterile discs were dipped into the culture of LAB isolates attached to the nutrient agar surface and incubated for 24 h at 37°C. The zone of inhibition was measured using a caliper. The experiment was conducted in triplicate [16]. Tolerance to simulated gastric juices was carried out to simulate the ability of isolates to survive in gastric conditions. Simulated gastric juice was prepared by dissolving 3 mg/mL pepsin (Merck) in physiological NaCl (0.9%) and setting it at pH 2.5. A portion of 0.1 mL of 18-hour-old isolate culture was inoculated and incubated overnight into each 10 mL of MRS broth at pH 6.5 and MRS broth at pH 2.5, with 3 mg/mL pepsin added for 4 h. Bile salt tolerance was determined by inoculating 0.1 mL of 18-hour-old isolate culture of isolates into 10 mL MRS broth supplemented with 0.3% bile salt (Merck) and incubated for 4 h at 37°C [17]. Bacterial growth was determined based on absorbance at a wavelength of 600 nm. The percentage of bacterial growth was measured by comparing the growth of bacteria in MRS broth (Himedia) pH 6.5 media with the growth of bacteria in MRS broth media conditioned according to the above treatment [18]. The resistance of isolates to simulated gastric juice and bile salt was measured in triplicate.

### 2.4. *α*-Glucosidase Inhibitor Activity Assay

Selected isolates were cultured in MRS broth media at 37°C for 24 h. The culture was centrifuged at 548 g for 15 min to separate the metabolite extract from the bacteria. Metabolites contained in the supernatant were tested for *α*-glucosidase enzyme inhibition activity [19]. Alpha-glucosidase inhibitor testing was determined based on the method of Susilowati et al. with slight modifications. An enzyme solution of 0.5 units/mL was prepared by dissolving 2695 mg *α*-glucosidase (Sigma-Aldrich) in 50 mL phosphate buffer at pH 7. After centrifuging the culture for 15 min at 548 g, the metabolite extract was separated from the bacteria. The activity of the metabolites in the supernatant to inhibit the *α*-glucosidase enzyme was evaluated. For 20 min, the reaction mixture containing 2 *μ*L metabolite extract in the supernatant, 48 *μ*L phosphate buffers (100 mM, pH 7), and 25 μL *α*-glucosidase enzyme (0.5 units/mL) was incubated at 37°C for 5 min. After incubation, 25 μL of substrate *p*-nitrophenyl *α*-D-glucopyranoside (Sigma-Aldrich) (20 mM) was added to the mixture. Further incubation for 15 min at 37°C was carried out and then 100 μL sodium carbonate/Na_2_CO_3_ 200 mM (Merck) was added to stop the reaction. The blank for the reaction was created by replacing the sample and enzyme with buffer. Similarly, the control was produced with a buffer instead of the sample. Furthermore, blank solutions were made for each sample by substituting an enzyme with a buffer. The antidiabetic drug acarbose was used as standard (10 mg/mL) for the assay. The percentage of *α*-glucosidase inhibition was calculated using the following formula [20]:

(1) $$\begin{array}{c} \text{Inhibition}\left(\%\right)=\left[\frac{\left({\text{Absorbance}}_{\text{control}}\left(A1\right)-{\text{Absorbance}}_{\text{sample}}\left(A1I\right)\right)}{{\text{Absorbance}}_{\text{control}}\left(A1\right)}\right]\times 100. \end{array}$$

### 2.5. DNA Extraction and 16S rRNA Gene Amplification

Bacterial isolates were cultured in MRS broth media and incubated at 37°C for 24 h. DNA of selected isolates was extracted using the Quick-DNA Fungal/bacterial miniprep kit. The DNA concentration obtained was 15.1 ng/*μ*L, with A260/280 and A260/230 values of 2 and 1.15, respectively. The 16S rRNA gene of LAB isolates was amplified using primers LbF-GGAATCTTCCACAATGGACG, LbR-CGCTTTACGCCCAATAAATCCGG. The PCR machine was programmed as follows: initial denaturation at 95°C for 5 min, followed by 30 cycles consisting of denaturation at 94°C for 45 s, hybridization at 60°C for 45 s, elongation at 72°C for 1 min, and final denaturation at 72°C for 10 min [21]. After PCR, the amplicon was separated by gel electrophoresis using 3% agarose and 1 μL loading dye with 5 μL PCR product and stained with gel red for gel documentation. PCR products were then sequenced using a DNA sequencer. Sequences of bacterial isolates were compared with sequences in GenBank (National Center for Biotechnology Information; https://www.ncbi.nih.gov) using the Basic Local Alignment Search Tool for nucleotide sequences. Phylogenetic tree construction was performed using the maximum likelihood method based on the Tamura-Nei model using Mega X software [22].

### 2.6. Whole Genomic Sequencing

Whole genomic sequencing of genomic DNA of potential isolates was performed using the Oxford Nanopore Technologies (ONT) sequencing platform with 1 GB output. For library preparation and sequencing, gDNA samples were used as input for library preparation using the Library Preparation Kit from ONT. gDNA was repaired using the final preparation enzyme mixture. The repaired DNA was ligated with ONT-compatible Adapters. The library was quantified with a Qubit Fluorometer before insertion into the Flow Cell. Sequencing was performed using GridION (ONT) until the desired results were achieved. GridION sequencing was operated by MinKNOW software. Base calling was performed using Guppy with high accuracy mode (HAC). Filtering was performed using Filtlong. Data transformation was performed using Samtools. Read quality was evaluated with Nanoplot. Readout correction and assembly were performed using Canu and Flye. The assembled sequences were polished with Racon four times and Medaka three times. Mapping was performed using minimap2. The quality of the assembled sequences was determined using Quast and Qualimap. To visualize genomic data, we used Proksee (https://proksee.ca/) [23].

### 2.7. Genomic Information Analysis of *Pediococcus acidilactici* Strain LBSU8

AMR genes were identified through ABRIcate software by selecting the NCBI Bacterial Antimicrobial Resistance Reference Gene Database. Virulence Factors Database (VFDB) software identified genes suspected of having virulence. Secondary metabolites were predicted using AntiSMASH 7.0 software (https://antismash.secondarymetabolites.org/) [24]. Detection strictness on the AntiSMASH website was set to “loose.” The databases and tools used in this study included Known Cluster Blast, MIBiG cluster comparison, Cluster Blast, Sub Cluster Blast, TFBS Finder, and Pfam and TIGRFam domains. To visualize homologous gene clusters, we used CAGECAT's *cblaster* tool (https://cagecat.bioinformatics.nl/). The complete genome of *Actinoplanes* was retrieved from the NCBI database and used as the query dataset to search for homologous sequences in other genomes. For a comparative visual analysis of these clusters, we applied the clinker module within CAGECAT. It generated publication-quality figures, illustrating gene arrangement and sequence identity within and across gene clusters from *Actinoplanes* as acarbose-producing bacteria. To visualize the amino acid sequences of the observed gene product, we used Clustal Omega multiple sequence alignment (https://www.ebi.ac.uk/jdispatcher/msa/clustalo).

## 3. Results

### 3.1. Biochemical Characterization of Isolates

A total of 6.7 × 10^6^ log CFU/mL of bacteria per mL *trites* were successfully isolated from the *trites*. From these bacterial colonies, 20 randomly selected isolates were further characterized. The purified colonies were then characterized. Characterization included Gram staining, morphology, catalase, hemolysis, and TSIA tests. The characterization results are shown in Table 1.

The hemolysis test results indicated that several isolates, including LBSU1, LBSU2, LBSU3, LBSU6, LBSU11, LBSU19, and LBSU20, exhibited *β*-hemolysis (clear zones observed around the colonies). Isolates showing *γ*-hemolysis, such as LBSU4 and LBSU7, were non-hemolytic, as no zones were observed. Based on these results, isolates with *β*-hemolysis, which indicate potential pathogenicity, were excluded from further experiments.

### 3.2. Bile Salt and Acid Tolerance

**Figure 1:**
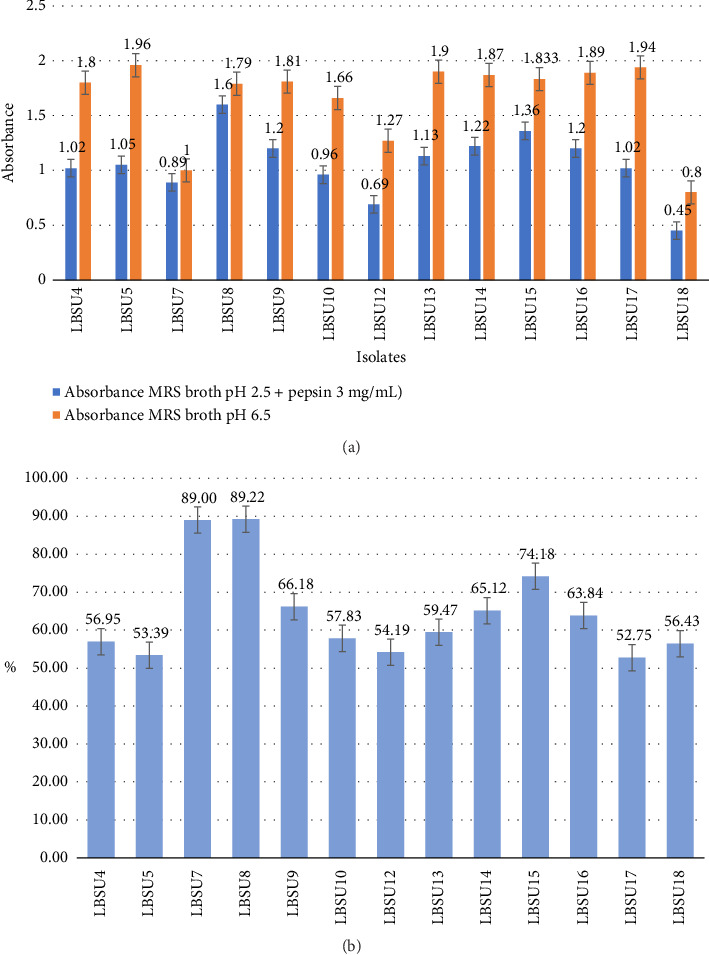
(a) The growth of bacterial isolates on MRS broth media (pH = 6.5) was compared with growth in simulated gastric juice (MRS broth pH 2.5 + 0.3% pepsin) incubated at 37°C for 4 h. (b) Growth percentage of bacterial isolates on simulated gastric juice media (MRS broth pH 2.5 + 0.3% pepsin).

Simulated gastric fluid was made by adjusting the pH of MRS broth to 2.5 and adding 0.3% pepsin. The growth of the isolate is indicated based on the absorbance value at *λ* = 600 nm. The survival ability of the isolate to grow in gastric juice is shown in Figure 1.

**Figure 2:**
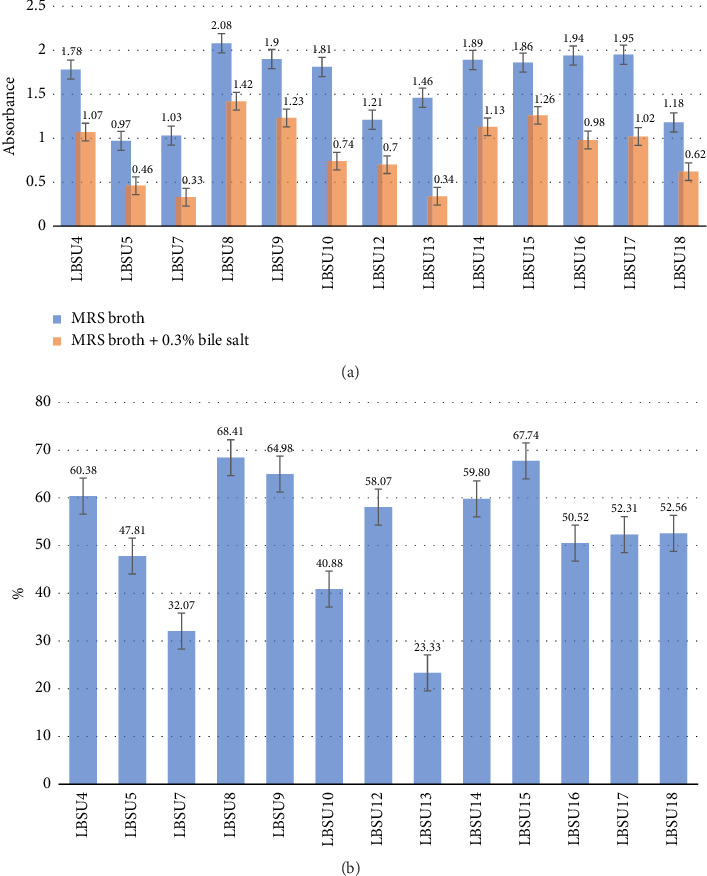
(a) The growth of bacterial isolates on MRS broth with 0.3% bile salt was compared with the growth of bacterial isolates on MRS without bile salt addition, incubated at 37°C for 4 h. (b) Growth percentage of bacterial isolates on MRS broth + bile salt 0.3% media.

From Figure 1, it can be seen that there is a decrease in isolate growth in MRS broth media compared to simulated gastric juice. The highest absorbance value was observed in isolate LBSU8, with a value of 1.6, indicating that this isolate has excellent tolerance to low pH. The isolates with the highest resistance to simulated gastric juice were LBSU8 and LBSU7, with growth percentages of 89% and 89.22%, respectively. This highlights their remarkable ability to survive in acidic conditions. The degree of acidity is a significant environmental parameter affecting microbial survival. The growth of the isolate is shown in Figure 2.

From Figure 2, it can be seen that there is a decrease in absorbance value in isolates grown in MRS broth media added with bile salt compared to isolates grown on MRS broth media alone. The highest absorbance was shown by isolate LBSU8 with an absorbance value of 1.42.  Figure 2(b) indicates that the isolate with the highest bile salt tolerance is isolated LBSU8, with a percentage of 68.41%.

### 3.3. Antimicrobial Activity

The antimicrobial activity of the isolates was tested against *E. coli* ATCC 25922 and *S. aureus* ATCC 25923 by the disc diffusion method. Amoxicillin was used as a positive control. The antimicrobial activity of the isolates is shown in Table 2.

Table 2 shows that isolate LBSU9 has the largest inhibition zone diameter; however, bacterial growth is observed within the clear zone of inhibition in a disc diffusion test, and it suggests the presence of inner colonies (ICs) that may indicate partial resistance or tolerance to the antibacterial agent being tested.

**Figure 3:**
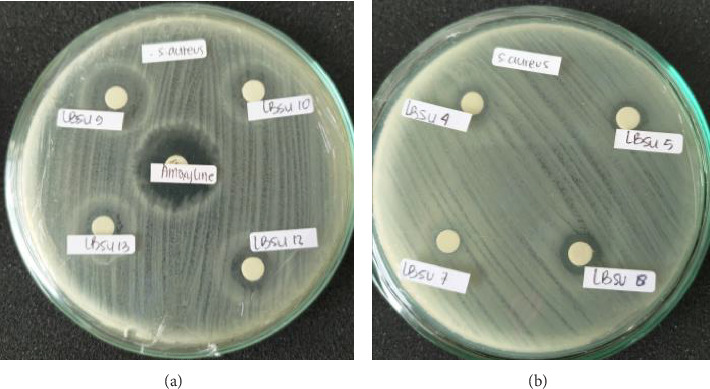
Antimicrobial activity of bacterial isolates against *S. aureus* ATCC 25923. (a) The yellow arrow indicates the antimicrobial activity of LBSU9. Inside the clear zone, there is still bacterial growth. (b) The black arrow indicates the antimicrobial activity of LBSU8.

This can be seen in Figure 3.

Isolate LBSU8 shows antibacterial activity categorized as susceptible with a reasonably strong inhibition zone diameter. Its diameter is 15.07 mm, which is even more potent than the diameter of the inhibition zone of amoxicillin, which is 11 mm. The isolates that were tested, LBSU9, LBSU12, LBSU13, LBSU15, and LBSU19, had intermediate antimicrobial activity. Interestingly, LBSU8 shows potent antimicrobial activity. Based on bacterial growth on MRS broth media conditioned to resemble the digestive tract and potent antimicrobial activity, isolate LBSU8 was selected for further identification and analysis.

### 3.4. Inhibitory *α*-Glucosidase Activity

The *α*-glucosidase inhibitor activity of isolate LBSU8 and acarbose is shown in Table 3.

LBSU8 isolate showed high *α* glucosidase inhibitor activity with a value of 98.6.

### 3.5. Molecular Identification of LBSU8

**Figure 4:**
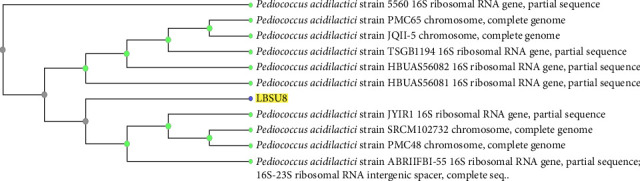
Phylogenetic tree analysis of LBSU8.

BLAST results of LBSU8 isolate against the NCBI database showed that it was identified as *Pediococcus acidilactici* strain LBSU8 with query cover and percentage identity reaching 100%. The sequence was registered to GenBank with accession number OR482644. Phylogenetic analysis of isolate LBSU8 (Figure 4) was determined using Neighbor-Joining by the NCBI Blast Tree Method.

### 3.6. Analysis of Whole Genome Sequence Assembly

**Figure 5:**
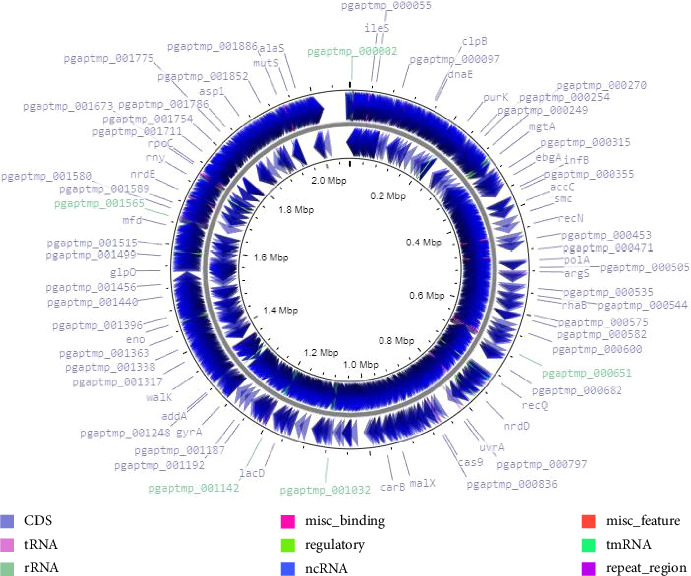
Visualization of whole genomic sequencing results of *Pediococcus acidilactici* strain LBSU8.

The visualization of the assembly results of *Pediococcus acidilactici* strain LBSU8 was analyzed using Proksee-Genome Analysis (https://proksee.ca/). Visualization is shown in Figure 5.

From Figure 5, it can be seen that the sequence length was 2 Mbp (2.071.271 bp). The quality of the assembled sequence was determined using Quast (Quality Assessment Tool for Genome Assemblies), as shown in Supporting file 1.

Bacterial virulence was identified based on the VFDB database using ABRicate software. The search results show that *Pediococcus acidilactici* strain LBSU8 is not virulent. AMR gene resistance was detected based on the NCBI Bacterial Antimicrobial Resistance Gene Database. The screening of antimicrobial-resistant genes was done using ABRicate with the NCBI Bacterial Antimicrobial Resistance Gene Database. Based on the in silico prediction of virulence factors using VFDB and antimicrobial-resistant genes using ABRicate, isolate LBSU8 did not carry any virulence and antimicrobial genes.

### 3.7. Secondary Metabolite Analysis

The genomic analysis of *Pediococcus acidilactici* strain LBSU8 revealed a division of its genomic region into 12 distinct gene clusters. These clusters are shown in Table 4, with each cluster associated with specific types of secondary metabolites.

**Figure 6:**
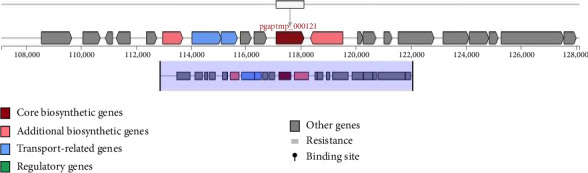
Biosynthetic gene clusters predicted by antiSMASH, highlighting the rfbB gene (pgaptmp_000121). The rfbB gene encodes dTDP-glucose 4,6-dehydratase. The functional domains of the genes within the clusters are indicated with color-coded arrows.

**Figure 7:**
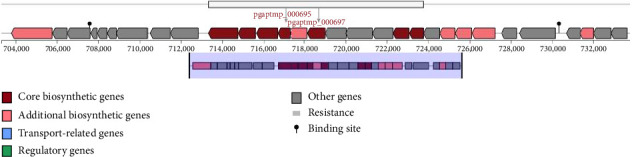
Biosynthetic gene cluster predicted by antiSMASH, highlighting the rfbB gene (pgaptmp_000695) and rfbA gene (pgaptmp_000697). The rfbB gene encodes dTDP-glucose 4,6-dehydratase, and the rfbA gene encodes glucose-1-phosphate-thymidylyltransferase. The functional domains of the genes within the clusters are indicated with color-coded arrows.

Using AntiSMASH software, we detected several genes in *Pediococcus acidilactici* strain LBSU8 and the secondary metabolites produced, associated with antimicrobial activity and its potential as *α*-glucosidase inhibitor. The antimicrobial activity of *Pediococcus acidilactici* strain LBSU8 is due to RiPP-type secondary metabolites produced in specific gene clusters such as phazolicin, thucin, nisin J, cinnamycin B, thuricin, duramycin, and nukacin ISK-1 and polyketide compounds including cepacin and jadomycin. In addition, other secondary metabolites have antimicrobial properties, such as corynecin I, II, and III. No secondary metabolites were found that directly showed properties as *α*-glucosidase inhibitors. Secondary metabolites in the second and sixth gene clusters are identified as extracellular polysaccharides (EPS) that are thought to have antidiabetic activity. The core biosynthetic gene cluster, specifically the 2nd and 6th gene clusters, encode the enzymes dTDP-glucose 4,6-dehydratase (RfbB) and glucose-1-phosphate thymidylyltransferase (RfbA), respectively, which may be involved in the synthesis of *α*-glucosidase inhibitors. The entire putative cluster genes of the second and sixth regions are shown in Figures 6 and 7.

**Figure 8:**
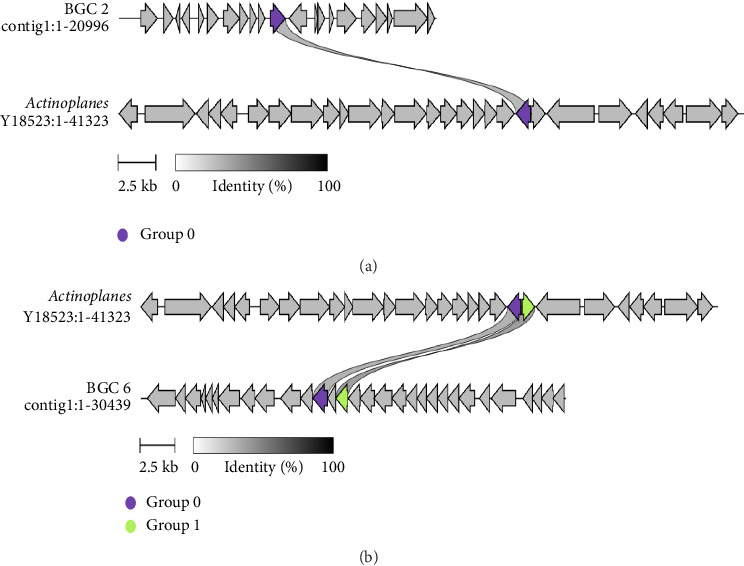
Comparative visual analysis of biosynthetic gene clusters (BCGs) in *Pediococcus acidilactici* strain LBSU8 and *Actinoplanes* sp. SE50/110. (a) BGC 2 of *Pediococcus acidilactici* shows similarity with the AcbB gene encoding dTDP-glucose 4,6-dehydratase in *Actinoplanes* sp. SE50/110. (b) BGC 6 of *Pediococcus acidilactici* demonstrates similarity with multiple genes in *Actinoplanes* sp. SE50/110, including the glucose-1-phosphate thymidylyltransferase RfbA (green arrow) and the dTDP-glucose 4,6-dehydratase AcbB (purple arrow), both of which are homologous to the rfbB gene in *Pediococcus acidilactici*. The analysis was performed using CAGECAT tool.

Comparative analysis of the biosynthetic gene clusters against *Actinoplanes* sp. *SE50/110* revealed significant sequence similarity between the dTDP-glucose 4,6-dehydratase (AcbB) and glucose-1-phosphate thymidylyltransferase (RfbA) genes of *Pediococcus acidilactici* strain LBSU8 and those in *Actinoplanes* sp. *SE50/110*. The visualization of BGC 2 and BGC 6 against *Actinoplanes* sp. SE50/110 complete gene is shown in Figure 8.

Further sequence alignment showed that the RfbB and AcbB genes share a 40.45% identity, while the identity between RfbA and AcbA is 49.48%. The homology between these genes can be seen in Supporting file 2.

We summarize the genes, gene products, and secondary metabolites in Table 5.

## 4. Discussion

The ability of potential probiotic microorganisms to tolerate intestinal bile salt is a critical component in their selection. It is crucial for their growth and survival in the gastrointestinal tract (GIT). For probiotics to successfully establish themselves in the gut of their host, they must possess resistance not just to bile salt but also to the acidic conditions of the GIT. Most external microbes perish upon entering the GIT due to the secretion of highly acidic gastric juice with a pH level of approximately 2.0. Therefore, probiotic bacteria must possess the ability to endure in very acidic settings (with a pH range of 1.0–3.0) and high concentrations of bile salt [25].

The digestive system's three main bactericidal agents are gastric secretions, HCl, and bile. Bile is another bactericidal agent found in the digestive system. Bile comprises many components: proteins, ions, pigments, cholesterol, and bile salt. Of these components, bile salt has been shown to protect against pathogenic bacteria. Among several criteria for selecting candidate probiotic strains of *Lactobacillus* spp., bile salt resistance is among the most critical selective criteria [26].

Probiotics, such as *Lactobacillus* and *Bifidobacterium*, have developed various mechanisms to resist bile salts, a key component of bile that can harm bacteria. These mechanisms include the efflux of bile salts or protons, modification of sugar metabolism, and prevention of protein misfolding [27]. The resistance of probiotics to bile salts can be influenced by their genomic variability, with some strains showing better survival and resistance to bile exposure [28]. Probiotics, particularly strains of *Lactobacillus* and *Bifidobacterium*, have developed various mechanisms to tolerate bile salts, which are crucial for their survival and functionality in the GIT. These mechanisms involve genetic, enzymatic, and structural adaptations that enable these microorganisms to withstand the harsh conditions posed by bile salts.

Comparative genomics has identified specific genes in *Lactobacillus* that are associated with bile salt tolerance. These genes are involved in systems such as the phosphotransferase system, two-component systems, carbohydrate metabolism, and ATP-binding cassette (ABC) transporters. Overexpression of these genes in host strains has been shown to enhance bile salt tolerance significantly [29]. In addition, LAB produce bile salt hydrolase that play a critical role in bile salt tolerance by hydrolyzing the amide bond between bile acids and amino acids. This process detoxifies bile and facilitates the incorporation of cholesterol into bacterial membranes [30].

*Pediococcus* species, particularly *Pediococcus acidilactici* and *Pediococcus pentosaceus*, have developed several mechanisms to survive the acidic conditions of the stomach, which is crucial for their potential as probiotics. These mechanisms include acid tolerance responses, production of protective proteins, and adaptive evolution strategies. *Pediococcus acidilactici* has shown improved acid tolerance through adaptive evolution. This process involves gradual exposure to low pH environments, which enhances the bacterium's ability to survive and maintain viability at pH levels as low as 4.0. However, further decreases in pH can significantly reduce lactic acid production, indicating a limit to this adaptation [31]. Similar to other enteric bacteria, *Pediococcus* species may utilize acid shock proteins to survive acidic conditions. These proteins help maintain cellular functions and protect against acid-induced damage [32].

The results of antimicrobial activity showed that isolate *Pediococcus acidilactici* strain LBSU8 showed the most potent activity against *S. aureus* and *E. coli*. Additionally, this strain is predicted to produce several antimicrobial compounds that effectively inhibit bacterial and fungal growth. These include non-ribosomal peptides (NRPs), ribosomally synthesized and post-translationally modified peptides (RiPPs), and polyketides [33]. Further analysis indicates that *P. acidilactici* strain LBSU8 is non-virulent and does not carry AMR genes, supporting its potential application as a safe and effective probiotic strain [34]. In previous research, it was reported that the culture supernatant of *P. acidilactici* has demonstrated strong antibacterial activity against avian pathogenic *E. coli*, with microencapsulation techniques enhancing its viability and effectiveness under gastrointestinal conditions [35]. In tarhana fermentation, *P. acidilactici* PFC69 significantly reduced the levels of *S. aureus*, indicating its potential as a bioprotective culture in cereal-based fermentations [36].

Several antimicrobial secondary metabolites were identified from *P. acidilactici* strain LBSU8. For instance, RiPPs, derived from precursor polypeptides modified by specialized enzymes, have gained interest as potential therapeutic agents, particularly as alternatives to conventional antibiotics in combating bacterial resistance [37]. Phazolicin, triculamin, alboverticillin, and microcin are some of the RiPP-type secondary metabolites produced in second gene cluster. Phazolicin is an extensively modified peptide that exhibits narrow-spectrum antibacterial activity against several leguminous plant symbiotic bacteria. There are 2 mechanisms of action of phazolicin, namely, inhibiting bacterial protein translation both in vivo and in vitro, as evidenced by the ability of phazolicin to reduce luciferase mRNA translation in vitro using *E. coli* S30 extract, and inhibiting the nascent peptide exit tunnel (NPET) [38]. Phazolicin can enter the cells of *S. meliloti* bacteria through two different pathways. The first pathway uses the peptide transporter, BacA, while the second uses the peptide transporter. Because phazolicin can enter through two distinct pathways, it is difficult for *S. meliloti* bacteria to become resistant to this antibiotic [39].

Microcins are low molecular weight (< 10 kDa), ribosomally produced, and highly stable antimicrobial proteins. Microcins form pores in the bacterial membrane, inhibiting aspartyl-tRNA synthetase, which is essential in protein synthesis and inhibits DNA gyrase and double DNA breaks. Some others block secondary RNA polymerase channels, interfere with transcription, and act on cytochromes to inhibit cellular respiration and interfere with cellular proton channels or ATP synthase [40].

The third gene cluster, identified through the Known Cluster Blast database, predicted to synthesize the lipopeptide fusaricidin B, which has antibacterial activity, particularly against *Bacillus subtilis*, and induces antibiotic-responsive genes. This compound is also produced by *Paenibacillus* sp. MS2379 is a potent biocontrol agent due to its antifungal activity. Fusaricidin B, C, and D, structurally similar to fusaricidin A, have been isolated from *Bacillus polymyxa* KT-8 and are active against fungi and Gram-positive bacteria [41]. Fusaricidin-type compounds have been shown to cause pores in mammalian cells' mitochondrial and plasma membranes, affecting their function and integrity [42]. Additionally, fusaricidin has been linked to the biosynthesis of fusarielins, a class of polyketides with antibacterial and antifungal properties [43].

In the fifth cluster gene, several antimicrobial secondary metabolite compounds are predicted to synthesize, including cepacin A and tubercidin. Cepacin A, a compound produced by *Burkholderia ambifaria*, is essential in regulating various phenotypes, including antifungal and antimicrobial properties [44]. Cepacin A and B, produced by *Pseudomonas cepacia*, have been found to have antibiotic properties, with cepacin A showing good activity against *Staphylococcus* and cepacin B showing excellent activity against *Staphylococcus* and some Gram-negative organisms [45]. Tubercidin, a potent antibiotic and antineoplastic agent, is produced by *Streptomyces tubercidicus* and has a unique structure that includes a 7-deazapurine core connected to a ribose moiety. Tubercidin exhibits antifungal and antiviral activity and is particularly effective against *Phytophthora capsica*. Tubercidin is also a potent cytotoxic agent against tumor cells [46].

Corynecin I, II, and III, produced by the 11th gene cluster, are antimicrobial metabolites with structures similar to chloramphenicol, differing only by the absence of two chlorine atoms. Corynecin III, like chloramphenicol, has broad-spectrum antimicrobial activity. Another antimicrobial metabolite, megacin, is produced by the 12th gene cluster. As a bacteriocin from *Bacillus megaterium*, megacin disrupts bacterial cytoplasmic membranes, leading to cell death, and inhibits nucleic acid, DNA, and protein synthesis, primarily targeting Gram-positive bacteria [47–49].

*Pediococcus acidilactici* has shown potential as an antidiabetic supplement in various studies. Zhao et al. reported that *Pediococcus acidilactici* is a probiotic that can counteract the effects of high glucose exposure [50]. Widodo et al. found that *Pediococcus acidilactici* reduced blood glucose levels and improved pancreatic beta cell function in diabetic rats [51]. In addition, certain strains of LAB have been found to have antioxidant and probiotic activities, which may contribute to the prevention and control of diseases associated with oxidative stress [52]. Our results show that *Pediococcus acidilactici* strain LBSU8 exhibits *α*-glucosidase inhibitor activity of up to 98.6%, which even exceeds the activity of acarbose, 97%.

Until now, the mechanism of production of *α*-glucosidase inhibitor compounds in probiotics, especially *Pediococcus acidilactici*, has never been reported. The presence of the enzyme dTDP-D-glucose 4,6-dehydratase in a *Pediococcus acidilactici* strain LBSU8 can be an indicator that the microbe has the potential to produce *α*-glucosidase inhibitor compounds such as acarbose. This enzyme is essential in the biosynthetic pathway, creating a critical acarbose molecule component. Acarbose formation is believed to involve the condensation of the C7-cyclitol component with dTDP-4-amino-4,6-dideoxy-D-glucose, the addition of maltose, and modifications of 5-epi-valiolone-7-P further down the pathway [53]. Aminocarbasugars, particularly valienamine, are significant carbapyranose derivatives that inhibit microbial oligosaccharide *α*-glycosidases. Valienamine is synthesized from sedoheptulose 7-phosphate, a key intermediate from the pentose phosphate pathway, which has two phases: the oxidative generation of NADPH and the non-oxidative interconversion of sugars. In the non-oxidative phase, transketolase catalyzes the production of sedoheptulose 5-phosphate, which cyclizes to form 2-epi-5-epi-valiolone, the precursor to acarbose [54]. Based on secondary metabolite mining from *Pediococcus acidilactici* strain LBSU8, the transketolase enzyme is synthesized by one of the additional antibiotic genes, the tkt gene.

The dominant secondary metabolites in the sixth and tenth gene clusters are capsular polysaccharides (CPS). Bacterial EPS can be classified as CPS, where the polysaccharide is tightly associated with the cell surface, or as mucous polysaccharides, where the polysaccharide is loosely associated with the cell. Previous studies have reported the effects of EPS from LAB on insulin-resistant HepG2 cells. Two common pathways examined were PI3k/AKT (phosphatidylinositol 3-kinase/protein kinase B) and AMPK (AMP-activated protein kinase) pathways, which have essential roles in coordinating anabolic and catabolic processes, especially in T2DM. EPS obtained from *Lactobacillus plantarum* H31 decreased *α*-amylase activity and increased gene expression of GLUT-4 (insulin-regulated glucose transporter type-4), AKT-2, and AMPK in insulin-resistant HepG2 cells under in vitro conditions. These genes increase glucose uptake in insulin-resistant cells through the insulin signaling pathway. The action of insulin in taking glucose from the blood into cells (hepatocytes) through the translocation of GLUT-4 vesicles to the plasma membrane is regulated by the PI3K/AKT signaling pathway. In insulin resistance, the average amount of insulin produced is insufficient to move glucose into cells. The leading cause of T2DM is insulin resistance, which involves failing to translocate GLUT-4 vesicles to the plasma membrane, inhibiting glucose consumption. AMPK is a critical switch in regulating glucose and lipid metabolism in the liver. Therefore, treatment with moderate doses of EPS H31 on insulin-resistant HepG2 cells increased the expression of GLUT-4, AMPK, and AKT-2, indicating that glucose consumption had been achieved [55].

## 5. Conclusion

This study successfully isolated 20 LAB from *trites*, a traditional Indonesian fermented food. Of these, LBSU8 showed the best probiotic characteristics, such as resistance to acid, bile salt, and antibacterial activity. LBSU8 also exhibited the best *α*-glucosidase inhibitor activity with a value of 98.6%. This isolate was identified as *Pediococcus acidilactici* strain LBSU8. The results of genomic sequencing analysis show that this isolate is not virulent and does not carry AMR genes. Analysis using AntiSMASH software revealed that LBSU8 produces antimicrobial secondary metabolites, primarily RiPPs such as phazolicin, triculamin, microcin, cinnamycin, and megacin. Additionally, other secondary metabolites include polyketide and saccharide. While no specific secondary metabolites inhibiting *α*-glucosidase were identified, enzymes like dTDP-glucose 4,6-dehydratase, transketolase, and glucose-1-phosphate thymidylyltransferase may contribute to this activity. Further studies are recommended to investigate the precise mechanism of *α*-glucosidase inhibition and assess the potential clinical applications of *Pediococcus acidilactici* strain LBSU8 as an antidiabetic agent.

## Figures and Tables

**Table 1:** Characterization of isolates based on Gram staining, catalase test, TSIA, and hemolysis.

No	Isolates	Characteristics					
		Gram staining	Morphology	Catalase	TSIA test	Gas production (CO_2_)	Hemolysis
1	LBSU1	+	Bacilli	Negative	A/A	−	*β*
2	LBSU2	+	Bacilli	Negative	A/A	−	*β*
3	LBSU3	+	Bacilli	Negative	A/A	−	*β*
4	LBSU4	+	Bacilli	Negative	A/A	−	*γ*
5	LBSU5	+	Bacilli	Negative	K/A	+	*γ*
6	LBSU6	+	Bacilli	Negative	A/A	−	*β*
7	LBSU7	+	Bacilli	Negative	A/A	−	*γ*
8	LBSU8	+	Cocci	Negative	A/A	−	*γ*
9	LBSU9	+	Bacilli	Negative	A/A	−	*γ*
10	LBSU10	+	Bacilli	Negative	A/A	−	*γ*
11	LBSU11	+	Bacilli	Negative	A/A	−	*β*
12	LBSU12	+	Bacilli	Negative	A/A	+	*γ*
13	LBSU13	+	Bacilli	Negative	A/A	−	*γ*
14	LBSU14	+	Bacilli	Negative	A/A	−	*γ*
15	LBSU15	+	Bacilli	Negative	A/A	−	*γ*
16	LBSU16	+	Bacilli	Negative	K/K	−	*γ*
17	LBSU17	+	Bacilli	Negative	A/A	−	*γ*
18	LBSU18	+	Cocci	Negative	K/K	−	*γ*
19	LBSU19	+	Cocci	Negative	A/A	−	*β*
20	LBSU20	+	Cocci	Negative	A/A	−	*β*

*Note:* A/A: acid/acid (fermentation of glucose, lactose, and/or sucrose with acid production throughout the medium). K/A: alkaline/acid (glucose fermentation only, producing acid at the bottom of the medium). K/K: alkaline/alkaline (no sugar fermentation). β: beta hemolysis (complete destruction of erythrocytes on blood agar medium). *γ*: gamma hemolysis (no hemolysis).

Abbreviation: TSIA, Triple Sugar Iron Agar.

**Table 2:** Antimicrobial activity of isolates against *E. coli* ATCC 25922 and *S. aureus* ATCC 25923.

No	Isolates/positive control	Diameter of inhibition zone (mm)	
		*E. coli* ATCC 25922	*S. aureus* ATCC 25923
1	LBSU4	9.33	9.03
2	LBSU5	8.2	10.53
3	LBSU7	5.97	0
4	LBSU8	15.07	11.03
5	LBSU9	17.07	20
6	LBSU10	10.1	12.5
7	LBSU12	6	12.5
8	LBSU13	6	11.5
9	LBSU14	10.5	14.5
10	LBSU15	14	13.5
11	LBSU16	7	0
12	LBSU17	7.07	7.5
13	LBSU18	0	0
14	Amoxicillin	11	24.93

*Note:* The diameter of the inhibition zone is expressed in millimeters (mm), measured using a caliper, and reflects the effectiveness of isolates in inhibiting bacterial growth.

**Table 3:** Inhibitory *α*-glucosidase activity of LBSU8 and acarbose.

Samples	Absorbance_control_ (A1)	Absorbance_sample_ (A11)	Inhibition (%)
LBSU8	4.529	0.061	98.6
Acarbose	4.324	0.1	97

**Table 4:** Division of the *Pediococcus acidilactici* strain LBSU8 genomic region into 12 gene clusters.

Region	Type	From	To
Region 1.1	Saccharide	16,321	39,772
Region 1.2	Saccharide	107,121	128,116
Region 1.3	Fatty acids	386,402	410,650
Region 1.4	Saccharide	520,253	541,242
Region 1.5	Saccharide	647,022	687,674
Region 1.6	Saccharide	703,326	733,764
Region 1.7	Saccharide	742,991	777,576
Region 1.8	Saccharide	1,081,736	1,111,800
Region 1.9	Saccharide	1,456,630	1,476,673
Region 1.10	Saccharide	1,562,539	1,591,081
Region 1.11	Fatty acids	1,665,712	1,686,680
Region 1.12	Saccharide	1,697,654	1,718,646

**Table 5:** Genes, gene products, and secondary metabolites of *Pediococcus acidilactici* strain LBSU8 associated with antimicrobial compound production and *α*-glucosidase inhibitor.

Cluster gene	Number of nucleotides	Core biosynthetic gene (s)	Additional biosynthetic gene	Gene product	Secondary metabolite (based on database MIBiG comparison or KnownClusterBlast)	Type
1	23.452				Pederin, pseudopederin, pederone	NRP, polyketide
2	20.996	rfbB		dTDP-glucose 4,6-dehydratase	Phazolicin, triculamin, alboverticillin, microcin L, microcin N, colicin V, RaxX, thatisin	RiPP
3	24.249				Oveidomycin	Lipopeptide
4	20.990					
5	40.653				Cepacin A, tubercidin	
6	30.439	rfbB	tkt, RfbA	dTDP-glucose 4,6-dehydratase, transketolase, glucose-1-phosphate thymidylyltransferase	Capsular polysaccharide, heteropolysaccharide	Saccharide
7	34.586				Strobilurin A, tunicamycin B1, cinnamycin B1, thuricin, duramycin, nukacin ISK-1, streptocollin, nisin J, venezuelin, birimositide	Polyketide, RiPP
8	30.065			Glycosyl transferase	Caprazamycin	Nucleoside, saccharide
9	23.044			Glycosyl transferase	Gaburedin	
10	28.543			Glycosyl transferase	Capsular polysaccharide, thusin, nisin J, plantazolicin	Saccharide, RiPP
11	20.969				Corynecin I, II, III	
12	20.993				Megacin, avermectin	RiPP, polyketide

## Data Availability

The data supporting this study's findings are available on request from the corresponding author. The data are not publicly available due to privacy or ethical restrictions.

## Supporting Information

Supporting Information 1 Supporting file 1: assembly statistic based on Quast.

Supporting Information 2 Supporting file 2: homology analysis of RfbB, AcbB, AcbA, and RfbA amino acid sequences.
