# Supplementary material for: Isolation and Characterization of Lactic Acid Bacteria From “Trites” Having the Ability to Produce α-Glucosidase Inhibitors
Source: Int J Microbiol. 2025 Jan 7;2025:8864668. doi: 10.1155/ijm/8864668 (PMC11732287; doi:10.1155/ijm/8864668)
Supplement: Supporting Information 1 — Supporting file 1: assembly statistic based on Quast. [file 8864668.f1.docx]

Supplementary file 1. Assembly Statistic based on Quast

| Category | Statistic |
| --- | --- |
| Aligned to "Ref" | 2044083 bp |
| Fragments | 1 |
| G+C content | 42,23 |
| Reads | 221224 |
| Mapped Reads (%) | 95,01 |
| Properly Paired (%) | 0 |
| Genome statistics |  |
| Genome fraction (%) | 90,31 |
| Duplication Ratio | 1,001 |
| Largest Alignment | 389747 |
| Total Aligned Length | 1847837 |
| NGA50 | 148643 |
| LGA50 | 5 |
| Reads Mapping |  |
| Mapped (%) | 99,86% |
| Properly Paired (%) | 0 |
| Singletons (%) | 0 |
| Misjoint Mates (%) | 0 |
| Avg. Coverage Depth | 437 |
| Coverage ≥1x (%) | 100 |
| **Misassemblies** |  |
| Misassemblies | 28 |
| Misassemblies contigs length | 2010590 |
| **Mismatches** |  |
| Mismatches per 100 kbp | 502,54 |
| Indels per 100 kbp | 16,31 |
| N's per 100 kbp | 0 |
| **Statistics without reference** |  |
| contigs | 2 |
| Total Length | 2071265 |
| Total Length (≥1000 bp) | 2071265 |
| Total Length (≥10.000 bp) | 2071265 |
| Total Length (≥50.000 bp) | 2071265 |
