# Supplementary material for: Isolation and Characterization of Lactic Acid Bacteria From “Trites” Having the Ability to Produce α-Glucosidase Inhibitors
Source: Int J Microbiol. 2025 Jan 7;2025:8864668. doi: 10.1155/ijm/8864668 (PMC11732287; doi:10.1155/ijm/8864668)
Supplement: Supporting Information 2 — Supporting file 2: homology analysis of RfbB, AcbB, AcbA, and RfbA amino acid sequences. [file 8864668.f2.docx]

Supplementary file 2. (a). Homology sequence between RfbB (pgaptmp_000121) and dTDP-glucose 4,6-dehydratase AcbB on *Actinoplanes sp.* SE50/110; (b) Homology between dTDP-glucose 4,6-dehydratase AcbA on *Actinoplanes sp*. SE50/110 with glucose-1-phosphate thymidylyltransferase RfbA of *Pediococcus acidilactici* strain LBSU8


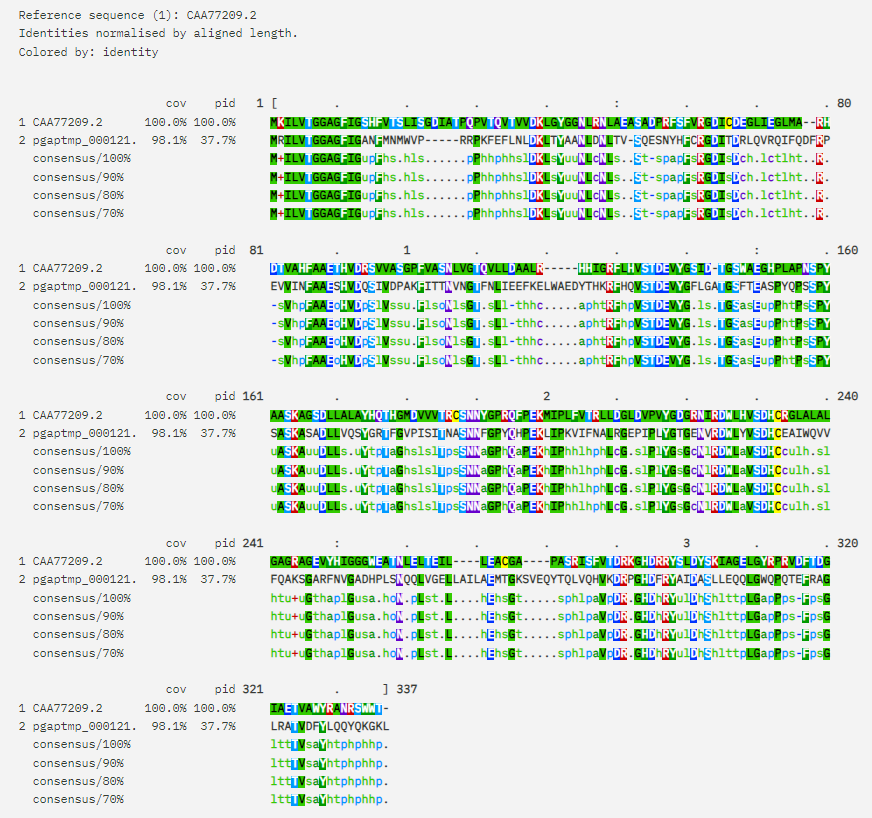


(a)


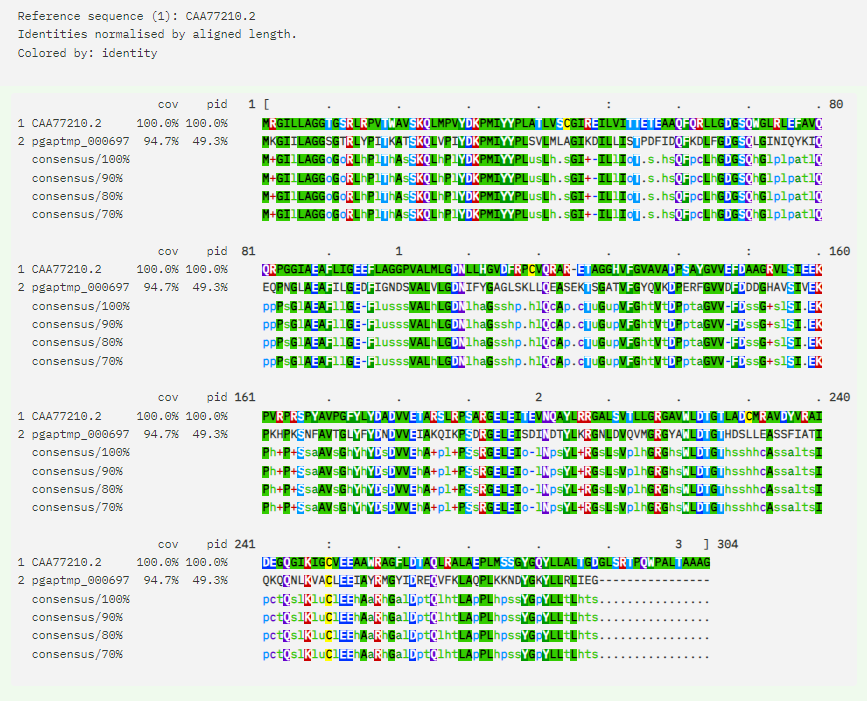


(b)

(a).Visualization of alignment of dTDP-glucose 4,6-dehydratase RfbB (pgaptmp_000121) and dTDP-glucose 4,6-dehydratase AcbB on *Actinoplanes sp.* SE50/110. B. Visualization of alignment of glucose-1-phosphate thymidylyltransferase RfbA (pgaptmp_000697) (b). Visualization of alignment of dTDP-glucose 4,6-dehydratase AcbA on *Actinoplanes sp*. SE50/110 with glucose-1-phosphate thymidylyltransferase RfbA of *Pediococcus acidilactici* strain LBSU8
